# Development and Characterization of a Rat Model of Blast Polytrauma and Hemorrhagic Shock for Evaluating Innate Immunotherapies During Prolonged Damage Control Resuscitation

**DOI:** 10.3390/cells15030250

**Published:** 2026-01-28

**Authors:** Milomir Simovic, Qingwei Zhao, Zhangsheng Yang, Leopoldo C. Cancio, Yansong Li

**Affiliations:** 1 Department of Organ Function Support, US Army Institute of Surgical Research, Fort Sam Houston, San Antonio, TX 78234, USA; msimovic@genevausa.org (M.S.); qingwei.d.zhao.ctr@health.mil (Q.Z.); zhangsheng.yang.ctr@health.mil (Z.Y.); leopoldo.c.cancio.civ@health.mil (L.C.C.); 2 The Geneva Foundation, Tacoma, WA 98402, USA; 3 Department of Surgery, University of Texas Health at San Antonio, San Antonio, TX 78229, USA

**Keywords:** blast injury, traumatic hemorrhage, innate immune response, multi-organ disfunction syndrome (MODS), prolonged damage control resuscitation (PDCR)

## Abstract

**Highlights:**

- This model recapitulates blast polytrauma-like multi-organ syndrome and supports traumatic hemorrhage therapeutic studies.
- Calls for the development of novel adjunctive therapies targeting innate immunity during prolonged damage control resuscitation to reduce organ injury and improve survival.

**What are the main findings?**

- The study demonstrated that the blast-plus-hemorrhage model triggers a rapid innate immune response, leading to inflammation-driven multi-organ damage.
- Plasma-Lyte A alone proves insufficient to break the vicious cycle of hypotension, hypoperfusion, and inflammation, highlighting the need for enhanced resuscitation strategies that address metabolic and innate immunity derangements in traumatic hemorrhage.

**What are the implications of the main findings?**

- The findings highlight complement-driven inflammation as a key driver of multi-organ injury, suggesting complement and innate immune pathways as promising therapeutic targets.
- This model mimics inflammation-driven multi-organ dysfunction syndrome in human polytrauma, enabling traumatic hemorrhage immunopathology and therapy studies.

**Abstract:**

Background: A major challenge in developing effective immunological damage-control therapies for traumatic hemorrhage (TH) is the lack of animal models that accurately reproduce the immune and pathophysiological responses observed in humans. In this study, we established a clinically relevant rat model that combines blast injury with hemorrhagic shock in a simulated prolonged damage control resuscitation environment. Methods: Male Sprague Dawley rats were anesthetized and subjected to moderate blast overpressure, followed by controlled hemorrhage equivalent to 40% of the estimated total blood volume. Animals then received hypotensive resuscitation with Plasma-Lyte A at twice the shed blood volume. Plasma-Lyte A was used in our study to correct hypovolemia and electrolyte imbalances, thereby helping to standardize the traumatic hemorrhage model. Results: Four of six rats in the blast-plus-hemorrhage (B + H) group survived the 25 h observation period. During resuscitation, mean arterial pressure remained markedly below baseline for at least 4 h. The B + H insult triggered a rapid innate immune response, characterized by elevated circulating HMGB1, terminal complement activation, and increased myeloperoxidase levels. Complement deposition (C4d, C5a, and C5b-9) was evident in lung tissue, accompanied by multi-organ histopathological injury, including pronounced inflammatory cell infiltration, hemorrhage, and cellular degeneration, apoptosis, or necrosis. Metabolic disturbances, including acidosis, hyperkalemia, and dilutional anemia, were also observed. Conclusions: Overall, this model reproduced key features of inflammation-driven multi-organ dysfunction syndrome seen in human polytrauma, supporting its utility for studying TH-related immunopathology and therapeutic interventions during prolonged damage control resuscitation.

## 1. Introduction

Traumatic hemorrhage (TH) remains the leading preventable cause of death in individuals under age 45 [1,2]. Fifty percent of TH-related deaths occur in the prehospital setting, either at the scene or during transport to the hospital, despite recent advances in the “golden hour” treatment protocols [3,4]. Current prehospital care of critically ill trauma patients is primarily supportive for TH patients and does not address the destructive effects of unchecked inflammation, which can progress to early multiple-organ dysfunction/failure (MODS/MOF) and lethal complications [5,6,7,8].

Traumatic hemorrhage causes tissue hypoperfusion, reduces oxygen delivery, and impairs energy production, leading to metabolic acidosis and cell death. In the early phase after TH, innate immune system activation, including damage-associated molecular patterns (DAMPs), complement cascade (ComC), and neutrophils, is thought to be a pivotal mediator in the development of early immune dysfunction. DAMPs (e.g., HMGB1) are released from mechanically injured, necrotic, stressed, and activated cells into the extracellular space. Recognition of DAMPs by pattern recognition receptors, such as intracellular nucleotide-binding oligomerization domain-like receptors and cell surface Toll-like receptors, induces inflammatory response [9]. Both TH itself and early TH-related therapeutic approaches (e.g., volume resuscitation, blood transfusion, surgical damage control, instrumentation) trigger systemic and local ComC activation, resulting in widespread inflammation and severe cytokine storm [5,6,9,10,11]. Neutrophils are among the main effector cells involved in early MODS/MOF following TH [12]. The neutrophil phenotype provides a unique snapshot of the immune response to trauma, representing the functional culmination of the complex cellular milieu observed in systemic inflammation, and can be activated by both DAMPs and ComC [12,13]. This innate immunity activation can result in systemic inflammatory response syndrome (SIRS) and endotheliopathy, which contribute to early MODS/MOF [5,6,7,8,9,10,11,12,13,14]. Late MODS/MOF is associated with compensatory anti-inflammatory syndrome and persistent inflammation/immunosuppression and catabolism syndrome [6,7,8,14]. MODS/MOF is strongly associated with mortality [15], and represents a leading cause of late mortality following severe trauma [16,17]. Mortality from MOF increases dramatically from 5% with single-organ failure to 90% or more when four or more organ systems fail [18].

Our work and that of others have shown that traumatic hemorrhage induces systemic inflammation [19,20,21,22,23,24,25,26]. Recent studies demonstrated an early “complementopathy” characterized by dysregulated ComC activation and consumption after explosive blast and/or TH [20,21,22,23,27,28,29,30]. This systemic ComC dysfunction appears to be a key driver of the pathological cascade following severe trauma. After major injury, the ComC, DAMPs, coagulation pathways, neutrophils, and cytokine/chemokine networks are rapidly mobilized as part of the innate immune “first line of defense.” While this response is protective in principle, excessive ComC activation acts as a major amplifier of systemic inflammation and is implicated in trauma-associated complications such as SIRS, sepsis, MOF, ischemia–reperfusion injury, and impaired wound healing [30,31,32,33,34,35]. Mortality after trauma may result either from the initial insult or from subsequent organ dysfunction, much of which stem from dysregulated early innate immune-inflammatory responses [5,6,7,36].

Prolonged damage control resuscitation (PDCR) is a US Department of Defense strategy for sustaining critically injured patients during extended evacuation delays of 24–72 h in austere and forward-deployed environments. PDCR focuses on preventing the “lethal triad” of coagulopathy, acidosis, and hypothermia through rapid hemorrhage control, aggressive use of blood products, permissive hypotension, and minimal crystalloid administration when definitive surgical care is not immediately accessible.

In this paper, we describe the development of a rat PDCR model that extends the principles of damage control resuscitation to a prolonged field care/preclinical setting (25 h) and recapitulates the inflammatory response and organ damage observed in severe injury, thereby establishing a platform for future mechanistic and innate immunity-targeted interventional studies.

## 2. Materials and Methods

### 2.1. Animal Study

This study was conducted in compliance with the Animal Welfare Act and its implementing regulations at an AAALAC-accredited institution, and in accordance with the principles of the *Guide for the Care and Use of Laboratory Animals*. The animal protocol and this study were reviewed and approved by the institutional review board of the US Army Institute of Surgical Research (approval code: A-16-019, approval date: 10 March 2016).

#### 2.1.1. Animal Surgical and Injury Procedures

**Figure 1:**
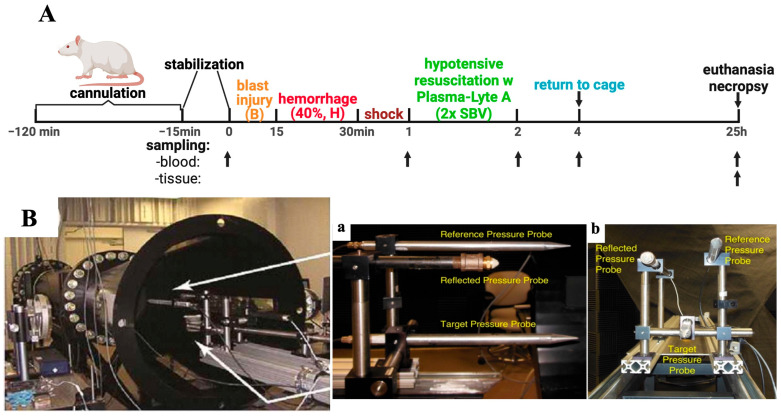
Schematic timeline and shock tube setup. (**A**) Experimental design. (**B**) Shock tube: side (**a**) and front (**b**) views showing the arrangement of pressure probes. Abbreviations: B, blast injury; H, hemorrhage; SBV, shed blood volume.

As outlined in Figure 1, male Sprague Dawley rats (300–350 g, 10–12 weeks old; Charles River Laboratories, Wilmington, MA, USA) were randomly assigned to either a combined-injury group (n = 6) or a sham control group (n = 5). Sham animals underwent identical anesthesia, handling, and procedural timing, but were not subjected to surgical procedures, blast overpressure (BOP), or hemorrhagic shock (Figure 1A).

Animals were anesthetized via intraperitoneal injection of ketamine/xylazine (60/5 mg/kg) and instrumented with catheters placed in the right carotid artery, left femoral artery, and femoral vein. After a 15-min period for surgical stabilization, BOP exposure (113 kPa, positive phase duration t_+_ = 3.2 ms) was delivered using a compressed air-driven shock tube (Applied Research Associates, Littleton, CO, USA) (Figure 1B). Each rat was positioned prone with its head facing the blast wave on a flat plastic mesh holder suspended between two horizontal rods. A second mesh was secured over the animal to stabilize its position, and the holder was mounted on a swing mechanism to allow partial recoil during blast exposure.

Fifteen minutes after BOP, animals underwent controlled hemorrhage of 40% of the estimated total blood volume over 15 min via the femoral arterial line. Following a 30- min shock period, hypotensive resuscitation was performed using Plasma-Lyte A at twice the shed blood volume (SBV), infused at 0.5 mL/min through the femoral venous catheter. For resuscitation, we used Plasma-Lyte A (pH 7.4, multiple electrolytes injection) for two reasons: (1) to model fluid resuscitation in the prehospital phase within an austere environment where blood products are unavailable, and (2) to create a traumatic hemorrhage model suitable for pathophysiological and immunological studies, as well as for evaluating diverse therapeutic approaches, including blood product transfusion. Plasma-Lyte A was not evaluated as a therapeutic intervention, but served as a tool for model standardization.

After blast exposure, anesthesia was maintained with 1–2.5% isoflurane. Three hours after shock, catheters were removed, vessels were ligated, and incisions were closed. Animals were then allowed to recover and were observed for 25 h in their home cages. Postoperative analgesia was provided with buprenorphine (0.1 mg/kg) administered subcutaneously at the nape of the neck using a 22-gauge, 0.5–1.5-inch needle every 8–12 h.

Throughout the experimental procedures, mean arterial pressure (MAP) was continuously monitored via the carotid catheter and recorded using a BIOPAC MP160 Data Acquisition and Analysis System (BIOPAC Systems, Inc., Goleta, CA, USA) until the animals recovered from isoflurane anesthesia.

#### 2.1.2. Blood Sampling and Necropsy

At the end of the 25 h experimental period, animals were euthanized with Fatal Plus (150 mg/kg). Necropsies were performed, and tissues were collected and fixed in either 10% formalin or 4% paraformaldehyde for histological and immunohistochemical analyses, respectively. Blood samples were obtained at five time points via the femoral or carotid arterial catheter or at euthanasia: post-cannulation (0), post-hemorrhagic shock (1 h), 1 h post-resuscitation (2 h), 2 h post-resuscitation (4 h), and 25 h after injury. Serum was collected after clotting, and EDTA plasma was prepared by centrifugation at 4000 rpm for 10 min. All serum and plasma aliquots were stored at −80 °C for later analysis.

### 2.2. Assays

#### 2.2.1. Blood Gas and Chemistry Laboratory Assays

Arterial blood gas analysis was performed at the bedside using an iSTAT 300-G analyzer (Abbott Point of Care Inc., Princeton, NJ, USA) with VetScan CG4+ and CG8+ cartridges (Abaxis Inc., Union City, CA, USA). The following parameters were measured: pH, pCO_2_, pO_2_, oxygen saturation, hematocrit, hemoglobin, sodium, potassium, chloride, ionized calcium, glucose, base excess/base deficit (BE/BD), and lactate. Baseline results were reviewed in consultation with the attending veterinarian.

#### 2.2.2. Analysis of Complement Functional Activity

Functional complement activation was measured by a hemolytic assay (CH50) [22,23,24,25,26]. Briefly, antibody-sensitized *Gallus gallus domesticus* red blood cells (Colorado Serum Company, Denver, CO, USA) were incubated with serial dilutions of serum samples in gelatin veronal buffer (GVB^++^ buffer, Complement Technology, Tyler, TX, USA) for 30 min at 37 °C. Following centrifugation, the absorbance of the supernatant was measured at 405 nm. The serum dilution producing 50% hemolysis was recorded as the CH50 value, and results were expressed as percentage change.

#### 2.2.3. Measurement of Plasma HMGB1 and Myeloperoxidase

Plasma myeloperoxidase (MPO) (Hycult Biotech Inc., Plymouth Meeting, PA, USA) and high mobility box 1 protein (HMGB1) (IBL-International, Baldwin Park, CA, USA) were quantified with enzyme-linked immunosorbent assay kits according to the manufacturers’ protocols.

### 2.3. Histological Examination and Tissue Injury Scoring

Formalin-fixed tissues (10%) were embedded in paraffin, sectioned coronally, and stained with hematoxylin and eosin (H&E). Five random fields were imaged at ×2, ×100, and ×400 magnifications using a slide scanner (Axio Scan.Z1, Carl Zeiss, Thornwood, NY, USA). A board-certified pathologist, blinded to the treatment groups, evaluated and scored the histological features according to previously described criteria [20,22,23,25,27,30].

### 2.4. Immunohistochemical Staining

Frozen tissue sections (5 µm) were processed for immunofluorescence staining. Sections were fixed in cold 4% paraformaldehyde for 10 min and then incubated overnight at 4 °C with primary antibodies against C4d (Hycult Biotech Inc., Plymouth Meeting, PA, USA), C5a (R&D Systems, Minneapolis, MN, USA), or C5b-9 (Hycult Biotech Inc., Plymouth Meeting, PA, USA). After washing with PBS, sections were incubated for 1 h at room temperature with the appropriate Alexa Fluor^®^ 555- or 594-conjugated secondary antibodies (Invitrogen, Carlsbad, CA, USA). Stained sections were mounted with ProLong^®^ Gold antifade reagent containing DAPI (Invitrogen, Carlsbad, CA, USA) and imaged at 200× or 400× magnification using an Olympus AX80 fluorescence microscope (Olympus, Center Valley, PA, USA). Negative controls were prepared by replacing the primary antibodies with the corresponding isotype controls.

### 2.5. Statistical Analysis

All data are expressed as means ± SEM. Group differences in inflammatory and organ injury parameters were analyzed using one- or two-way ANOVA, followed by Tukey’s post hoc multiple comparison test relative to baseline. For non-parametric datasets, Friedman repeated-measure ANOVA on ranks was applied. Post- hoc pairwise comparisons were conducted using the Holm–Šidák method or Tukey’s test with Bonferroni correction, as appropriate. A *p*-value < 0.05 was considered statistically significant.

## 3. Results

### 3.1. Blast Wave Parameters, Systemic Complement Activation, Plasma HMGB1, and Myeloperoxidase Levels After Blast Injury and Hemorrhagic Shock (B + H)

**Figure 2:**
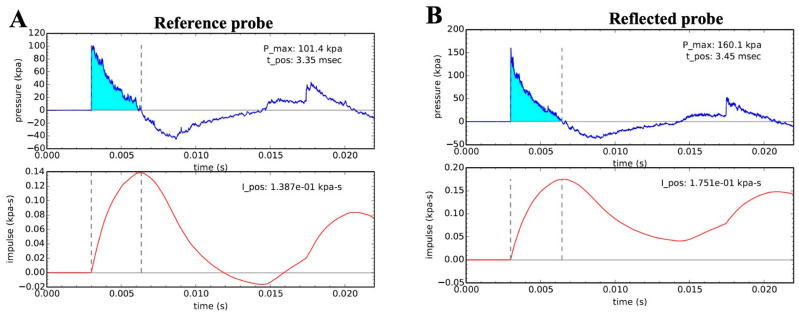
Representative shock waveforms. (**A**) Friedlander (top) and specific impulse (bottom) waveforms recorded by the reference probe. (**B**) Friedlander (top) and specific impulse (bottom) waveforms recorded by the reflected probe.

**Figure 3:**
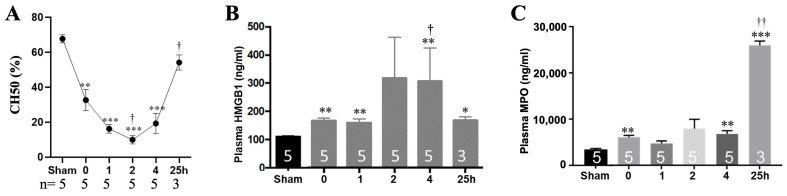
Systemic innate inflammatory response following blast injury and hemorrhage. Serum complement hemolytic activity (CH50, (**A**)) and plasma HMGB1 (**B**) and MPO (**C**) levels were quantified using a CH50 assay and ELISA, respectively. * *p* < 0.05, ** *p* < 0.01, *** *p* < 0.001 versus sham; ^†^ *p* < 0.05, ^††^ *p* < 0.01 versus baseline (0).

The shock tube generated consistent and reproducible open-field blast parameters (Figure 2 and Table 1). Surgical cannulation alone elicited terminal complement activation (TCA) (Figure 3A) as well as increased plasma HMGB1 (Figure 3B) and (MPO (Figure 3C). B + H provoked a more pronounced and rapid inflammatory response, with TCA peaking at 2 h post-injury before gradually returning toward baseline (Figure 3A). Plasma HMGB1 levels also rose at 2 and 4 h after injury relative to baseline, with statistical significance reached only at 4 h (Figure 3B). Plasma MPO showed a dramatic 10-fold elevation at 25 h (Figure 3C), indicating sustained neutrophil priming and activation, likely mediated by complement-driven inflammation.

### 3.2. Hemodynamic and Blood Chemistry Changes After Blast and Hemorrhage

**Figure 4:**
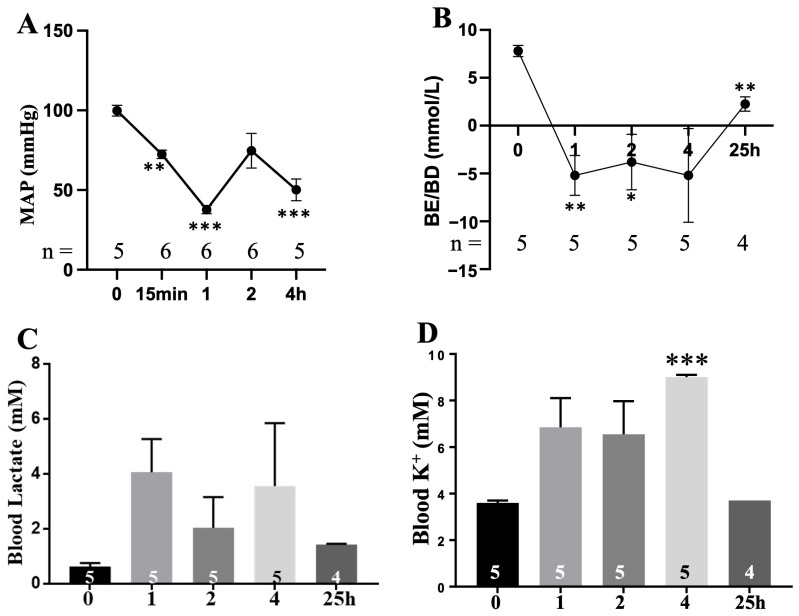
Hypovolemic shock, metabolic acidosis, and hyperkalemia after blast injury and hemorrhage. (**A**) Mean arterial blood pressure (MAP); (**B**) base excess (BE); (**C**) blood lactate; (**D**) blood potassium. * *p* < 0.05, ** *p* < 0.01, and *** *p* < 0.001 versus baseline (0).

**Figure 5:**
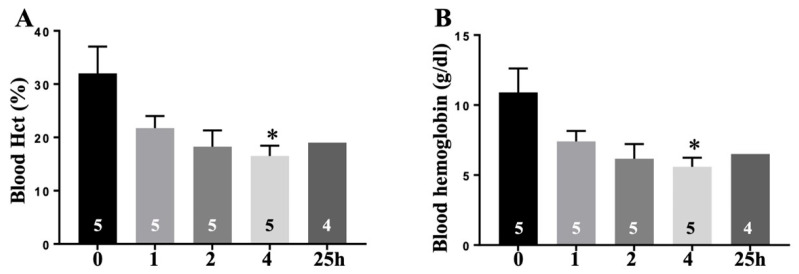
Dilutional anemia following blast injury and hemorrhage. Blood hematocrit (Hct, (**A**)) and hemoglobin (Hb, (**B**)) levels were measured using an i-STAT CHEM8+ analyzer. * *p* < 0.05 versus baseline (0).

The average MAP in injured animals fell from approximately 100 mmHg to 73 mmHg after blast exposure, dropped further to 38 mmHg following hemorrhage and the hypovolemic shock phase, and then recovered to about 70 mmHg after Plasma-Lyte A resuscitation, but not to normotension. MAP then declined again to ~50 mmHg within 2 h of resuscitation (Figure 4A). Blood base excess/base deficit (BE/BD) remained below zero throughout hemorrhagic shock and for an extended period thereafter, with only minimal recovery by 25 h, indicating a sustained tissue hypoperfusion. These findings suggest that Plasma-Lyte A at twice the shed blood volume did not rapidly or effectively correct tissue hypoperfusion (Figure 4B). Lactate levels demonstrated a biphasic increase, but showed no statistically significant differences in injured animals across the observation period (Figure 4C). Potassium levels increased between 1 h and 4 h post-injury, reaching statistical significance at the 4 h time point (Figure 4D). A significant reduction in hematocrit and hemoglobin was observed at 4 h, consistent with dilutional anemia secondary to the compensatory fluid shift and fluid resuscitation (Figure 5A,B).

Two of six injured animals died following resuscitation, whereas all sham animals survived 25 h, indicating a survival rate of approximately 60–70% in this model. The non-survivors exhibited severe hypotension (MAP < 40 mmHg) after Plasma-Lyte A infusion and ultimately died under refractory shock conditions.

### 3.3. Pulmonary Generation and/or Deposition of Complement Split Products in Response to Traumatic Hemorrhage

**Figure 6:**
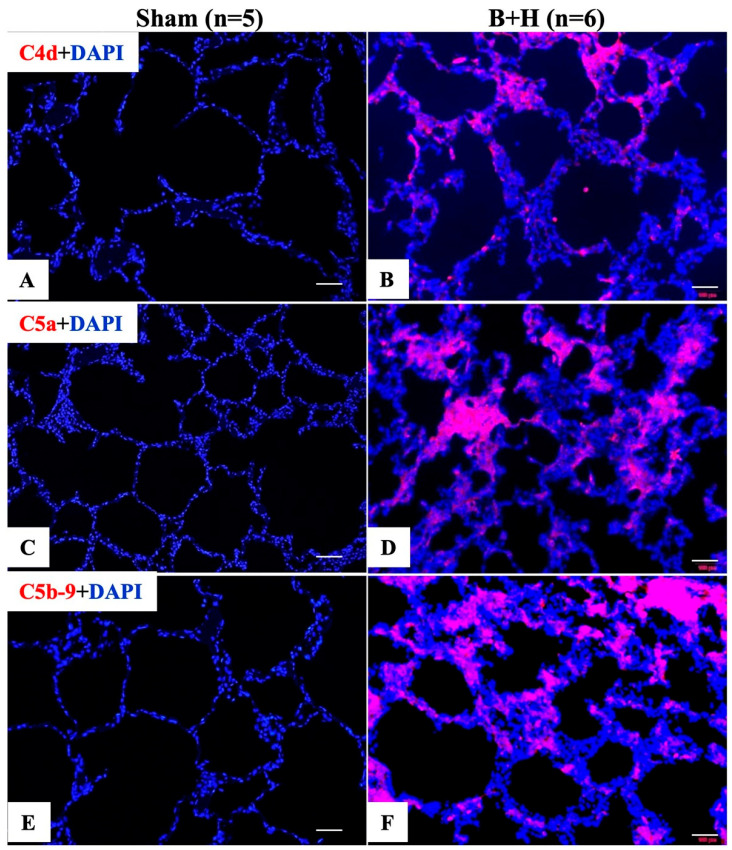
Pulmonary complement protein levels following blast injury and hemorrhage. Frozen lung sections were stained for C4d (**A**,**B**), C5a (**C**,**D**), and C5b-9 (**E**,**F**) using specific antibodies. Original magnification = 200×; scale bar = 100 μm.

Immunohistochemical analysis revealed strong pulmonary deposition of complement activation fragments C4d (Figure 6B), C5a (Figure 6D), and the terminal membrane attack complex C5b-9 (Figure 6F), localized primarily to alveolar septal cells, infiltrating inflammatory cells, and injured lung parenchyma. These complement deposits were absent in sham controls (Figure 6A,C,E).

### 3.4. Multiple-Organ Damage

**Figure 7:**
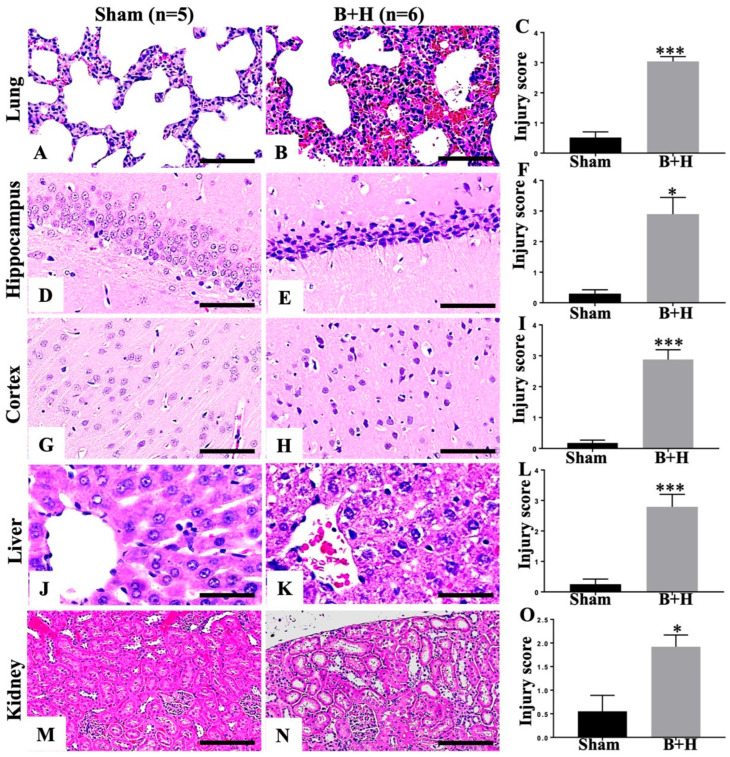
Multiple-organ damage following blast injury and hemorrhage. Histological alterations were assessed in H&E-stained paraffin sections. Representative histopathology and injury scores are shown for lung ((**A**–**C**), 400×, scale bar = 100 μm), hippocampus ((**D**–**F**), 400×, scale bar = 100 μm), cortex ((**G**–**I**), 400×, scale bar = 100 μm), liver ((**J**–**L**), 400×, scale bar = 100 μm), and kidney ((**M**–**O**), 200×, scale bar = 200 μm). Data are presented as means ± SEM and were analyzed using the Mann–Whitney U test. * *p* < 0.05, *** *p* < 0.001 versus sham.

Histopathological evaluation using hematoxylin and eosin (H&E) staining and corresponding injury scores revealed marked multi-organ damage in the injured group (Figure 7B,E,H,K,N) compared with sham controls (Figure 7A,D,G,J,M). In the lungs, injury was characterized by disruption of normal alveolar architecture, extensive intra-alveolar hemorrhage, septal edema, and prominent neutrophil infiltration within both the septa and alveolar spaces (Figure 7B). In the hippocampus, the injured animals exhibited significant neuronal loss and neurodegeneration across the CA1, CA2, CA3, and dentate gyrus regions, evidenced by reduced neuronal density, pyramidal cell degeneration, cell body shrinkage, nuclear pyknosis, loss of nucleoli and Nissl substance, and intense eosinophilic cytoplasm (Figure 7E). The cerebral cortex also showed substantial gray matter injury, including neuronal swelling, nuclear condensation, cytoplasmic vacuolization or spongiform change, and associated vasogenic edema with perivascular vacuolization (Figure 7H). Liver sections demonstrated hepatocellular degeneration, featuring condensed nuclei, pale eosinophilic cytoplasm, and evidence of cell death consistent with both apoptosis and necrosis (Figure 7K). Mild renal injury was also observed, including loss of tubular brush borders, hydropic degeneration, dilation of Bowman’s space with hyaline material accumulation, and vascular congestion (Figure 7N). Semiquantitative scoring of injury severity on histology further validated these observations (Figure 7C,F,I,L,O).

## 4. Discussion

In our study, combined blast and severe hemorrhage triggered a rapid innate immune response characterized by TCA, elevated plasma HMGB1 levels, and increased MPO. Accumulation of activated complement proteins (C4d, C5a, C5b-9) was observed in lung tissue, accompanied by tissue hypoperfusion and multiple-organ histopathological damage, including inflammatory cell infiltration, hemorrhage, cellular degeneration, apoptosis, and necrosis across multiple organs.

TH remains a significant clinical challenge, particularly due to the complex interplay between immune dysregulation and MODS/MOF. To address the lack of clinically relevant animal models, we developed a rat model combining blast injury with hemorrhagic shock that recapitulates the immune and pathophysiological responses observed in human polytrauma [23,24,25,26,29,37,38,39]. The findings provide valuable insights into the mechanisms underlying inflammation-driven multiple-organ damage and highlight the critical limitations of current resuscitation strategies.

The model successfully reproduced key features of human TH, including sustained hypotension, tissue hypoperfusion, metabolic disturbances, and innate inflammation-driven MODS [23,24,25,26,37,38,39]. However, vascular catheterization itself induced baseline complement activation, confounding the effects of combined blast and hemorrhage. This study design did not allow us to distinguish complement activation caused by cannulation from that induced by the combined injury. In a subsequent improved approach, cannulated rats were allowed to recover for 5–7 days before undergoing the combined injury. Under this protocol, CH50 in injured rats decreased 15 min after blast injury due to complement consumption, reached its nadir before resuscitation, and returned to baseline 11 h post-injury [23].

Animals and humans can compensate for significant hemorrhage through various neural and hormonal mechanisms. Modern trauma care enables patients to survive when these adaptive compensatory mechanisms become overwhelmed. Permissive hypotensive resuscitation using restrictive fluid therapy has become standard practice to increase systemic blood pressure to suboptimal levels while avoiding exacerbation of uncontrolled bleeding. We used Plasma-Lyte A rather than normal saline because the latter has been associated with increased acute kidney injury and mortality [40,41,42]. Plasma-Lyte A closely mimics human plasma in electrolyte content, osmolality, and pH, and provides additional buffer capacity through anions such as acetate and gluconate, which are metabolized to bicarbonate, CO_2_, and water. Acetate, a bicarbonate precursor, offers several advantages over other commonly used bases such as lactate. Plasma-Lyte A corrects volume and electrolyte deficits while addressing metabolic acidosis. The absence of calcium in Plasma-Lyte A allows for safe co-administration with most drugs, blood, and blood products [40,41,42]. As a balanced crystalloid, Plasma-Lyte A appears superior to normal saline and is suitable for prehospital care.

Hemodynamic analysis revealed that Plasma-Lyte A resuscitation at twice the shed blood volume restored mean arterial pressure (MAP) to post-blast levels, but failed to achieve normotension or effectively correct tissue hypoperfusion. Persistent acidosis, hyperkalemia, and dilutional anemia further underscored the inadequacy of this approach in reversing metabolic derangements. The survival rate of 60–70% in the injured group highlights the severity of the insult and the need for improved therapeutic strategies.

Acute severe trauma along with DAMPs and pathogen-associated molecular patterns and early therapeutic approaches (blood product transfusion, ECLS devices, damage control surgery, ventilation, volume resuscitation, etc.) rapidly activate the intravascular innate immune system consisting of the plasma cascade systems (complement, coagulation, and the contact system, blood cells, and endothelial cells), ultimately leading to trauma-induced thromboinflammation, MODS/MOF, and mortality [5,6,7,8,9,10,11,13,29,31,32,33,34,35,36].

The innate immune response was characterized by elevated circulating HMGB1, TCA and sustained neutrophil priming, as evidenced by a dramatically increased plasma MPO levels. Histopathological evaluation revealed extensive multi-organ damage: pulmonary injury, neurodegeneration in the hippocampus and cerebral cortex, hepatocellular degeneration, and mild renal injury. The observed neuronal loss and neurodegeneration in the hippocampus and cerebral cortex align with the neurological deficits often seen in human TH patients. Similarly, liver and kidney injuries reflect the systemic impact of TH and the limitations of current resuscitation strategies in mitigating organ damage.

Our previous work and that of others demonstrated that: (1) early elevated blood levels of activated complement proteins (e.g., C3a, C4d, C5a, C5b-9, Bb), HMGB1, and MPO at the scene or on admission are observed in both combat casualties and civilian trauma patients, correlating positively with each other and associating with SIRS, injury severity score, tissue hypoperfusion, coagulopathy, endotheliopathy, increased blood product and crystalloid requirements, mechanical ventilation, prolonged hospital stay, MOF, and mortality [23,24,25,26,29,37,38,39]; (2) traumatic injury induces immediate and sustained leukocytosis with sterile trauma instantly triggering nuclear DNA release with evidence of neutrophil extracellular trap formation and immediate post-injury increases in both pro- and anti-inflammatory cytokines—patients who developed MODS had significantly higher injury severity scores and lower base excess at admission [43]; (3) moderate BOP with or without hemorrhage in rats leads to metabolic acidosis, increased local C3/C5b-9 deposition, systemic TCA and cytokine storm, elevated expression of MPO and intracellular adhesion molecule 1 in the lungs, enhanced leukocyte infiltration in the brains, and upregulation of TNF-α in the brain tissue [20,22,23,27,30]; and (4) severe hemorrhage in rats results in significant deposition of C3, C5a, and C5b-9 in the lung and small intestines, with C3 and C5b-9 colocalizing with pulmonary and intestinal vasculature [44].

Mortality after trauma may result from either the initial insult or subsequent MODS/MOF, much of which stem from dysregulated early innate immune-inflammatory responses [5,6,7,8,9,10,11]. Despite its recognized importance, the mechanisms governing complement activation after trauma and its full clinical relevance remain incompletely understood, and no therapy currently exists to rectify trauma-induced complement dysfunction. Notably, we recently demonstrated that early administration of the C5 inhibitor nomacopan [23] and the HMGB1 inhibitor CX-01 [25] significantly reduced morbidity and mortality 25 h after blast and hemorrhage in rat models.

The acetate and gluconate buffers in Plasma-Lyte A may normalize serum lactate levels through systemic buffering without resolving underlying tissue hypoperfusion or cellular metabolic dysfunction. This dissociation between systemic laboratory values and tissue-level metabolic distress represents a critical limitation when relying on lactate as a resuscitation endpoint during balanced crystalloid administration. The biphasic lactate pattern observed in our data, though not statistically significant, may reflect transient tissue hypoxia that is biochemically masked at the systemic level by buffering effects. These findings underscore the need for complementary markers of tissue perfusion to more accurately assess metabolic status during PDCR with buffered crystalloids. Such markers could include base deficit, lactate clearance kinetics using advanced techniques (e.g., ^13^C-labeled lactate with mass spectrometry), or regional perfusion monitoring with near-infrared spectroscopy.

The lack of statistical significance in lactate and HMGB1 levels, despite the severity of the traumatic hemorrhage, likely reflects multiple contributing factors. First, the buffering capacity of Plasma-Lyte A may have played a role in reducing lactate accumulation and indirectly decreased passive HMGB1 release from stressed cells. Second, individual animals may have different capacities of hepatic lactate clearance (Cori cycle) and HMGB1 release. Third, the limited sample size may have reduced statistical power to detect differences in lactate and HMGB1 levels between groups.

Dobson et al. explain early and late deaths following hemorrhagic trauma as a systems failure, presenting a unified systems hypothesis of trauma based on four pillars: (1) CNS-cardiovascular coupling, (2) endothelial–glycocalyx function, (3) mitochondrial integrity, and (4) immune dysregulation [7,8]. Maintaining this systems homeostasis after TH may switch the injury phenotype toward a survival phenotype.

Pathophysiological changes in polytrauma patients have recently been considered through four pathogenetic, interactive cycles, adding soft tissue injury to the mortality triad (hemorrhage/hypoxemia, coagulopathy, and hypothermia) [45,46]. Each of these states can trigger a systemic inflammatory response, leading to generalized endothelial damage [45,46].

In the present study, we observed a significant decrease in BE/BD (indicating tissue hypoperfusion) over 25 h and substantial deposition of complement activation fragments (C4d, C5a, and C5b-9) in pulmonary tissue, associated with intra-alveolar hemorrhage, septal edema, and neutrophil infiltration. Together, these findings indicate that complement-driven inflammation plays a central role in the development of TH-related lung injury.

The reduced hematocrit and red blood cell (RBC) count (Figure 5) are most likely attributable to (a) complement deposition-induced RBC deformability and/or (b) redistribution of extracellular fluid from interstitial to intravascular compartments as an initial compensatory response to blood loss. Accumulating evidence indicates that trauma triggers complement activation, leading to deposition of activated complement proteins such as C3d, C4d, and C5b-9 on RBC surfaces. This deposition reduces RBC deformability, impair capillary transit, and oxygen exchange, and is associated with increased nitric oxide production. Together, these changes represent a key mechanism of post-traumatic RBC dysfunction that may contribute to organ injury [47,48].

## 5. Conclusions

This rat model provides a robust platform for investigating TH-related immunopathology. Future research should prioritize approaches targeting complement activation and other innate immune pathways to mitigate multi-organ damage and improve survival outcomes in TH during prolonged field care.

## 6. Limitations

This study has several following limitations. First, the stabilization period between the surgical procedure and injury induction was insufficient, as indicated by significant TCA and elevated plasma HMGB1 and MPO levels at baseline compared with sham controls. To better isolate the effects of the trauma, reduce experimental variability, and improve data reliability, future studies should incorporate an adequate post-surgery recovery period to allow physiological and immunological parameters to normalize or alternatively employ a minimally invasive surgical approach.

Second, although complement functional activity was assessed using CH50, measurement of additional circulating complement slit products (e.g., C3a, C4d, C5a, C5b-9, Bb) and broader complement pathway-specific markers would provide more detailed insights into complement activation mechanisms.

Third, more comprehensive analysis of renal, hepatic, and brain functional biomarkers is required in future studies. Fourth, both male and female animals should be recruited in future preclinical studies. Fifth, the small number of subjects may have reduced the statistical power to detect meaningful differences between groups in the 25 h endpoint analyses. Therefore, these findings should be interpreted with caution given the limited number of animals included. Future studies with larger samples would enable more comprehensive statistical analyses and provide greater confidence in the reliability and external validity of the findings.

Finally, beyond systemic HMGB1 and MPO measurements, evaluation of their local expression and spatial distribution in tissue using immunohistochemistry, Western blotting, and PCR would further clarify their roles in organ-specific injury. These complementary analyses are planned as part of our ongoing efficacy studies and are expected to yield deeper mechanistic understanding.

## Figures and Tables

**Table 1:** Blast wave parameters.

	Reference			Reflected		
	P0 (kPa)	t_+_ (ms)	I (kPa-ms)	P0 (kPa)	t_+_ (ms)	I (kPa-ms)
B + H (n = 6)	101.17 ± 1.09	3.31 ± 0.03	135.95 ± 0.94	154.17 ± 2.32	3.46 ± 0.01	170.87 ± 1.56

Notes: B + H, blast injury + hemorrhagic shock; I, impulse; P0, peak pressure; t_+_, positive-pressure phase duration.

## Data Availability

The original contributions presented in this study are included in the article material. Further inquiries can be directed to the corresponding author.

## Abbreviations

The following abbreviations are used in this manuscript.

B + H	blast injury and hemorrhagic shock
BOP	blast overpressure
CH50	hemolytic assay
ComC	complement cascade
DAMPs	damage-associated molecule patterns
H&E	hematoxylin and eosin
HMGB1	high mobility group box 1 protein
MAP	mean arterial pressure
MODS	multiple-organ dysfunction syndrome
MOF	multiple-organ failure
MPO	myeloperoxidase
PDCR	prolonged damage control resuscitation
SBV	shed blood volume
SIRS	systemic inflammatory response syndrome
TCA	terminal complement activation
TH	traumatic hemorrhage
